# ATM and ATR Expression Potentiates HBV Replication and Contributes to Reactivation of HBV Infection upon DNA Damage

**DOI:** 10.3390/v11110997

**Published:** 2019-10-31

**Authors:** Anastasiya Kostyusheva, Sergey Brezgin, Ekaterina Bayurova, Ilya Gordeychuk, Maria Isaguliants, Irina Goptar, Felix Urusov, Anastasiya Nikiforova, Elena Volchkova, Dmitry Kostyushev, Vladimir Chulanov

**Affiliations:** 1 National Medical Research Center of Tuberculosis and Infectious Diseases, Ministry of Health, Moscow 127994, Russia; Seegez@mail.ru (S.B.); vladimir.chulanov@rcvh.ru (V.C.); 2 Institute of Immunology, Federal Medical Biological Agency, Moscow 115522, Russia; 3 NF Gamaleya Research Center of Epidemiology and Microbiology, Moscow 123098, Russia; p1ngv1n2009@yandex.ru (E.B.); lab.gord@gmail.com (I.G.); maria.issagouliantis@rsu.lv (M.I.); 4 Chumakov Federal Scientific Center for Research and Development of Immune-and-Biological Products of Russian Academy of Sciences, Moscow 108819, Russia; 5 Sechenov First Moscow State Medical University, Moscow 119146, Russia; az@rcvh.ru; 6 Department of Pathology, Riga Stradins University, LV-1007 Riga, Latvia; 7 Department of Microbiology, Tumor and Cell Biology, Karolinska Institutet, SE-171 76 Stockholm, Sweden; 8 Izmerov Research Institute of Occupational Health, Gene Engineering and Biotechnology, Moscow 105275, Russia; probirka@list.ru (I.G.); flanger.fx@mail.ru (F.U.); utkina.anastasia@gmail.com (A.N.); 9 Central Research Institute of Epidemiology, Moscow 111123, Russia

**Keywords:** HBV reactivation, replication, DNA damage, ATM, ATR, phosphorylated H2AX, yH2AX, shRNA, CRISPR/Cas9, CRISPRa, dCas9

## Abstract

Chronic hepatitis B virus infection (CHB) caused by the hepatitis B virus (HBV) is one of the most common viral infections in the world. Reactivation of HBV infection is a life-threatening condition observed in patients with CHB receiving chemotherapy or other medications. Although HBV reactivation is commonly attributed to immune suppression, other factors have long been suspected to play a role, including intracellular signaling activated in response to DNA damage. We investigated the effects of DNA-damaging factors (doxorubicin and hydrogen peroxide) on HBV reactivation/replication and the consequent DNA-damage response. Dose-dependent activation of HBV replication was observed in response to doxorubicin and hydrogen peroxide which was associated with a marked elevation in the mRNA levels of ataxia-telangiectasia mutated (ATM) and ATM- and RAD3-related (ATR) kinases. Downregulation of ATM or ATR expression by shRNAs substantially reduced the levels of HBV RNAs and DNA. In contrast, transcriptional activation of ATM or ATR using CRISPRa significantly increased HBV replication. We conclude that ATM and ATR are essential for HBV replication. Furthermore, DNA damage leading to the activation of ATM and ATR transcription, results in the reactivation of HBV replication.

## 1. Introduction

Hepatitis B virus (HBV) causes acute or chronic hepatitis B virus infection (CHB) [1]. HBV infection affects over 2 billion people worldwide; around 250 million people are chronically infected and 1 million die annually due to liver cirrhosis, hepatic decompensation, and hepatocellular carcinoma [2]. Modern antivirals effectively inhibit virus replication and substantially decrease the risks of unfavorable HBV infection outcomes [3]. Comorbidities in patients with CHB often require additional interventions, such as cancer chemotherapy, immunosuppressive therapies after liver transplantation, treatment of viral hepatitis C in HBV-HCV-co-infected individuals which may trigger an abrupt reactivation of HBV infection [4,5]. In patients undergoing chemotherapy and therapy with immunosuppressive drugs, treatment may result in lethal HBV reactivation [6,7,8]. This turns HBV reactivation into a serious clinical problem posing a major threat to global public health. 

Therapies that can result in HBV reactivation can be divided into several major groups based on their mode of action, namely: DNA damage, TNF-α inhibition, B-cell depletion, histone deacetylase inhibition, chemokine or integrin inhibition, kinase inhibition, and proteasome inhibition [5]. The precise mechanisms of HBV reactivation upon administration of different agents remain largely unknown, but has been linked either to the impairment of immune responses (TNF-α inhibitors, B-cell depleting agents, chemokine and integrin inhibitors, kinase inhibitors, proteasome inhibitors) or activation of transcription of HBV covalently closed circular DNA (cccDNA) (histone deacetylase inhibitors) [5]. Several lines of evidence indicate that HBV reactivation may be related to oxidative stress, DNA damage/DNA damage response and interconnected alterations in the intracellular signaling and cell cycle progression in the infected cells [9,10,11,12,13,14,15]. 

The DNA damage response (DDR) involves activation of a series of kinases, including the phosphatidyl inositol 3-kinase-like kinases (PIKKs), ataxia-telangiectasia mutated (ATM), ATM- and RAD3-related (ATR), and catalytic subunit of DNA-dependent protein kinase (DNA-PKc) [16]. Phosphorylation of PIKK target proteins results in the formation of phosphorylated H2AX foci or yH2AX; foci of p53-binding protein (53BP1, one of the key proteins of DNA double-stranded break [DSB] repair); phosphorylated ATM; and accumulation of p53 in the nucleus, all indicative of DNA damage [16]. Activated ATM and ATR phosphorylates their downstream substrates Chk2 and Chk1, respectively [17], which in turn affects different biochemical pathways of the cell, including cell cycling and gene transcription [18]. DDR includes sensing, initiation, amplification, and implementation of cellular responses to DNA damage, ultimately leading to either the repair of damaged DNA, or cell senescence and death [19]. 

Viruses require DDR to effectively replicate in the infected cells [20]. They may recruit ATM, ATR, and DNA-dependent protein kinase catalytic subunit (DNA-PKcs) and their interacting partners, as well as other DDR proteins, including MRE11A and RAD51 [20]. DDR has long since been recognized as an important player in HBV transcription and replication [21]. We and others have previously demonstrated that the livers of patients with CHB and with HBV/hepatitis D virus co-infection harbor yH2AX foci [22,23], a reliable marker of activated DDR [24]. In vitro studies demonstrated that HBV infection activates DDR, resulting in increased levels of ATR and phosphorylated checkpoint kinase 1 (Chk1) [25]. Knocking down ATR decreases HBV transcription and replication, mainly due to reduced Chk1 phosphorylation. Additionally, DDR has been reported to enhance activity of HBV cccDNA promoters both in vitro and in vivo [10]. Importantly, cytoplasmic HBV X (HBx) protein, a key factor of HBV cccDNA transcription, induces endoplasmic reticulum (ER) stress and DNA damage by activating the ATM-checkpoint kinase 2 (Chk2) signaling cascade, resulting in increased HBV transcription and replication [26]. On the contrary, inhibitors of ATM and ATR kinases (caffeine and theophylline) or of Chk1 (UCN01) suppress HBV replication and HBV-related pathogenesis [25]. These findings suggest an important role of DDR signaling in the natural HBV life cycle. In line with this, chemotherapeutic agents which induce DNA damage and activate DDR, such as doxorubicin [9] and cisplatin [13] which trigger p53, p21, ATR, and Chk1 signaling, may trigger reactivation of HBV infection. 

Despite this accumulated evidence, the precise mechanisms involved in HBV reactivation upon DNA damage, as well as the roles of individual DDR components in the HBV replication, remain largely unknown. 

To address the role of DDR in HBV reactivation and replication, we analyzed HBV replication in cells after DNA damage induced by doxorubicin, a DNA-damaging drug used to treat various malignancies [27], or hydrogen peroxide (H_2_O_2_) [28]. We show that doxorubicin and H_2_O_2_ cause severe DNA damage, which, in turn, upregulates the transcription of ATM and ATR and stimulates HBV replication. We have transiently knocked down ATM and ATR with respective shRNA, and found that suppression of ATM and ATR transcription suppressed HBV replication. Conversely, transcriptional activation of ATM or ATR using the CRISPRa technique [29] potentiates HBV replication, mimicking the effects of DNA-damaging agents. These findings indicate that ATM and ATR play an important role in the reactivation of HBV replication switching it upon DNA damage. Further studies of the basic mechanisms behind HBV reactivation are of crucial importance for the correct management of patients with CHB with respect to the choice of medical interventions (transplantation, chemotherapy or other), and also for the design and application of new antiviral drugs.

## 2. Materials and Methods

### 2.1. Cell Culture

Human HEK-293T (Invitrogen, Thermo Fisher Scientific, Waltham, MA, USA), HepG2, HepG2-1.1merHBV, and HepG2-1.5merHBV (kindly provided by Dieter Glebe, University of Giessen, Germany) cell lines were maintained in complete DMEM with 10% FBS (Thermo Fisher Scientific, Waltham, MA, USA), 2 µM l-glutamine (Sigma Aldrich, St. Louis, MO, USA), and 1% penicillin/streptomycin (Sigma Aldrich, St. Louis, MO, USA). HepG2-1.1merHBV is an inducible cell line with a 1.1-mer HBV genome regulated by a tet-on CMV promoter, HepG2-1.5merHBV produces HBV constitutively; both cell lines have been described previously [30,31]. HepG2-1.1merHBV cells were supplemented with 100 ng/mL doxycycline (Sigma Aldrich, St. Louis, MO, USA) to activate HBV replication. Doxycycline was removed after 24 h of incubation, and cells were washed twice with PBS and cultured in complete medium before harvest. HEK-293T cells were transfected with HBV-encoding plasmid containing 1.1merHBV under CMV promoter [32] using polyethylenimine (Sigma Aldrich, St. Louis, MO, USA). Recombinant cccDNA was produced using minicircle technology (System Biosciences, Palo Alto, CA, USA) in a ZYCY10P3S2T *E. coli* bacterial recombination system generating circular HBV cccDNA genome with elimination of DNA plasmid backbone, and used to transfect HepG2 cells as was described previously [33].

### 2.2. Chemicals

Doxorubicin (Sigma Aldrich, St. Louis, MO, USA) was dissolved in DMSO (Sigma Aldrich, St. Louis, MO, USA) and aliquots were stored at −80 °C. H_2_O_2_ (3% solution in water) was stored at room temperature.

HepG2-1.1MerHBV, activated HepG2-1.1merHBV, and HepG2 cells transfected with HBV-encoding plasmid were treated with doxorubicin or H_2_O_2_ for 1 h at concentrations provided in Table 1. Next, cells were washed twice with PBS and either immediately harvested for analysis, or incubated for an additional 24 h.

### 2.3. Isolation of Nucleic Acids

At harvest, the culture medium was discarded, and cells were washed twice with PBS and lysed in AmpliSens Riboprep lysis buffer (AmpliSens Biotechnologies, Moscow, Russia). Nucleic acids were isolated using the AmpliSens Riboprep kit (AmpliSens Biotechnologies, Moscow, Russia) according to the manufacturer’s instructions. RNA was isolated as described previously. Briefly, nucleic acids were treated with RNase-free DNase I (New England Biolabs, Ipswich, MA, USA) for 30 min at 37 °C, purified using the AmpliSens Riboprep kit, and reverse-transcribed using AmpliSens Reverta-FL (AmpliSens Biotechnologies, Moscow, Russia). HBV cccDNA was isolated via the HIRT procedure as described by Cai et al. [34], followed by treatment with plasmid-safe ATP-dependent DNase (Epicentre, Illumina Inc., Madison, WI, USA) for 12  h at 37 °C and inactivating the enzyme at 72 °C for 15 min. Secreted HBV DNA was analyzed in a cell culture supernatant; viral DNA was isolated using the AmpliSens Riboprep kit (AmpliSens Biotechnologies, Moscow, Russia) according to manufacturer’s instructions and PCR-quantified with specific primers and probes. 

### 2.4. PCR Analysis

A real-time quantitative polymerase chain reaction (qPCR) was performed using fluorescent probes TaqMan or SYBRGreen dye (Invitrogen, Thermo Fisher Scientific, Waltham, MA, USA).

PCR targeting viral pregenomic RNA (pgRNA) amplified pre-core 3.5 kb and pregenomic 3.5 kb HBV transcripts, surface mRNA (S-RNA) detecting both 3.5 kb transcripts, 2.4 kb and 2.1 kb HBV RNAs and ATM, ATR, DNA-PK, MRE11a, and RAD51 mRNAs were assessed in relation to the mRNA of the GAPDH reference gene. Levels of the total intracellular and secreted HBV DNA and cccDNA were normalized to the content of β-globin genomic DNA. Specific sets of primers and probes are presented in Table 2. Relative expression levels were calculated via the ^ΔΔ^Ct method.

### 2.5. Knockdown of DDR Factors

The human U6 promoter amplified by PCR from pLX-sgRNA (AddGene plasmid #60662) and shRNA-encoding oligonucleotides were cloned into pGEM-T Easy (Promega, Madison, WI, USA). ShRNA targets were (1) ATM: 5′-TAGTGTTAGTGATGCAAACGA-3′; (2) ATR: 5′-TACTGACCAATTGAAACTCTA-3′; and (3) scrambled control: 5′-CCTAAGGTTAAGTCGCCCTCG-3′. The efficacy of shRNA knockdowns and their effects on HBV were initially tested in HEK-293T cells co-transfected with pGEM-T Easy carrying shRNA-encoding oligonucleotides and plasmid encoding HBV by polyethylenimine. Transfection of shRNAs into HepG2-1.1merHBV cells was performed using Lipofectamine 3000 (Thermo Fisher Scientific, Waltham, MA, USA) according to the manufacturer’s instructions. Cells were transfected, washed with PBS twice 2 days post transfection (pt), and harvested 5 days pt. 

### 2.6. ATM and ATR Overexpression

A CRISPR activation tool (dCas9-p300) was used to transcriptionally activate ATM and ATR. For this, sgRNAs targeting corresponding genes were designed in the CHOPCHOP sgRNA designing tool. The following sgRNAs target sequences 5′-ACAGTTCCGAAGGCGAACGGG-3′ and 5′-CGTGCGTCGGTCAAGTTTCC-3′ were used for ATM and ATR activation, correspondingly. The dCas9-p300mut protein with inactivated p300 activity (Addgene #61358) was expressed in cells together with the non-targeting sgRNA 5′-GGGGCCACTAGGGACAGGAT-3′ as a mock control. PCR-products encoding sgRNA under the control of the U6-promoter were synthesized as described previously using a 2-step mutagenic PCR with a Q5 high-fidelity polymerase and purified using the Qiagen gel extraction kit (QIAGEN, Hilden, Germany) [31]. HepG2 cells were transfected using Lipofectamine 3000 with a mix containing (1) recombinant cccDNA synthesized using minicircle technology (System Biosciences, Palo Alto, CA, USA); (2) a plasmid encoding dCas9p300 (pcDNA-dCas9-p300 Core; Addgene #61357); and (3) a PCR product encoding gRNA. Cells were incubated for 72 h and used for analysis of ATM and ATR expression by qPCR as described in Section 2.4.

### 2.7. Immunocytochemistry

HepG2-1.1mer cells were seeded on glass coverslips and fixed in 4% paraformaldehyde for 10 min. Next, the coverslips were washed 3 times in Tris-HCl (50 mM, pH 8.0), incubated for 30 min with a blocking buffer (0.02% of Triton X-100, 10% horse serum, and 150 mM NaCl in Tris-HCl, 50 mM, pH 8.0), and incubated with primary mouse monoclonal anti-yH2AX antibodies (ab26350) and rabbit polyclonal anti-53BP1 (ab175933, Abcam, Cambridge, UK) antibodies at room temperature for 1 h. The cells were washed 3 times for 5 min in a washing buffer (0.02% of Triton X-100 and 200 mM NaCl in Tris-HCl, 50 mM, pH 8.0), then incubated with secondary Alexa Fluor 488 goat anti-rabbit IgG antibodies (ab205718, Abcam, Cambridge, UK), Alexa Fluor 594 goat anti-mouse antibodies (ab150116, Abcam, Cambridge, UK), and Hoechst33342 (to visualize the nucleus; 1/10,000; ab228551, Abcam, Cambridge, UK) at room temperature for 1 h. The coverslips were washed 3 times for 5 min in a washing buffer and mounted with a Fluoroshield reagent (Abcam, Cambridge, UK). Images were captured using a Leica DMI6000 microscope with 100× immersion objectives. yH2AX and 53BP1 foci were counted visually or using ImageJ (NIH, Bethesda, MD, USA). Foci were quantified for at least 200 cells in each experimental group. The results are presented as a mean number of foci per cell in randomly selected areas from three independent experiments (each dot corresponds to a mean number of foci in a selected area). The research was done using the equipment of the Core Centrum of Institute of Developmental Biology, RAS (Moscow, Russia).

### 2.8. Quantification of HBsAg 

Cell-conditioned media were harvested and filtered through 0.2 µm filters (Corning Inc., New York, NY, USA) to remove cell debris. An HBV surface antigen (HBsAg) analysis in culture medium was performed using the Abbott Architect HBsAg kit (Abbott Laboratories, Abbott Park, IL, USA). 

### 2.9. Statistical Analysis 

Values were expressed as means ± standard deviation (SD) of triplicate experiments in the GraphPad Prism software (GraphPad Software Inc., San Diego, CA, USA). Students’ t-test or one-way ANOVA with Tukey’s HSD post hoc test were used to compare variables and calculate *p*-values to determine statistically significant differences in means.

## 3. Results

### 3.1. DNA Damage Promotes HBV Replication

**Figure 1:**
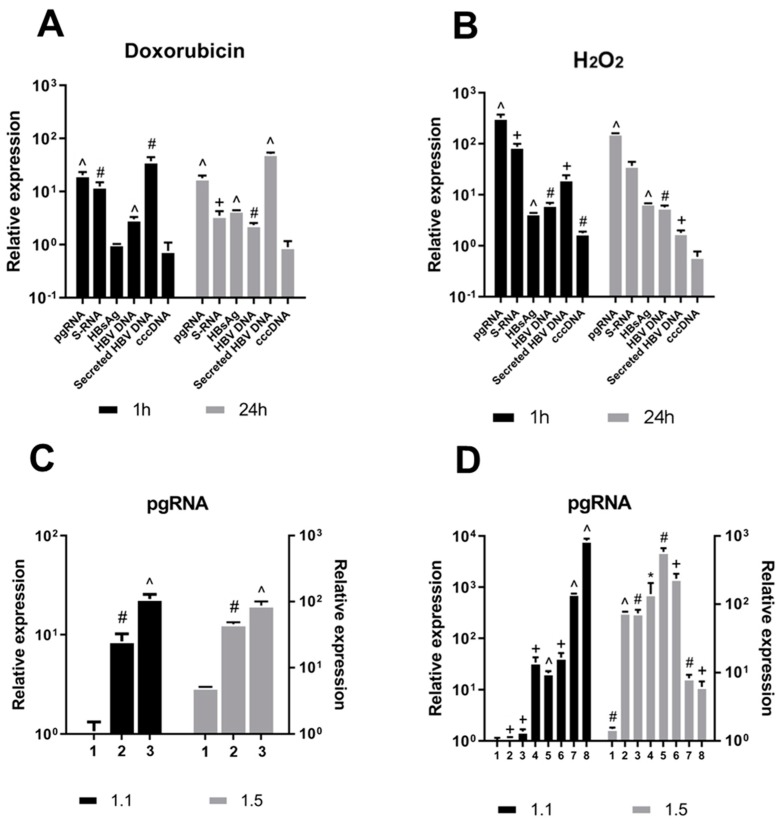
Upregulation of HBV replication by DNA-damaging agents. Alterations in HBV intermediates (pgRNA, S-RNA, intracellular HBV DNA, secreted HBV DNA and secreted HBsAg) upon (**A**) doxorubicin and (**B**) H_2_O_2_ treatment in HepG2-1.1merHBV cells. Data are relative to mock-treated control. Effects of increasing doses of (**C**) doxorubicin and (**D**) H_2_O_2_ on pgRNA levels in HepG2-1.1merHBV cells (1.1) and HepG2-1.5merHBV cells (1.5). X-axis represents different doses of doxorubicin and H_2_O_2_ as specified in Table 1. The results were reproduced in at least 3 independent experiments. Asterisks indicate statistically significant differences in means compared to control. * *p* < 0.05, ^+^ *p* < 0.01, ^#^ *p* < 0.001, ^^^ *p* < 0.0001.

Treating HepG2-1.1merHBV cells with either doxorubicin or H_2_O_2_ (according to Figure S1) rapidly increased HBV transcription and replication (Figure 1A,B). HBV transcription increased 15–20-fold after doxorubicin treatment and over 100–300-fold after H_2_O_2_ treatment. HBV transcription was accompanied by increase in the levels of intracellular and secreted HBV DNA and HBsAg (Figure 1A,B). Levels of HBV intermediates increased as early as 1 h post treatment and remained elevated 24 h after removal of the chemicals.

Next, we studied the effects of different doses of doxorubicin and H_2_O_2_ on the levels of HBV pgRNA in HBV-replicating cell lines HepG2-1.1merHBV and HepG2-1.5merHBV (Figure 1C,D). The highest dose of doxorubicin tested (0.5 µM, Table 1) induced 20- to 100-fold upregulation of HBV transcription in both cell lines (Figure 1C). H_2_O_2_ concentrations as low as 2–4 mM increased HBV pgRNA levels up to 8000-fold compared to untreated cells (Figure 1D). However, in HepG2-1.1merHBV cells, application of H_2_O_2_ in concentrations higher than 7 mM led to a decline in the levels of HBV pgRNA (Figure 1D).

**Figure 2:**
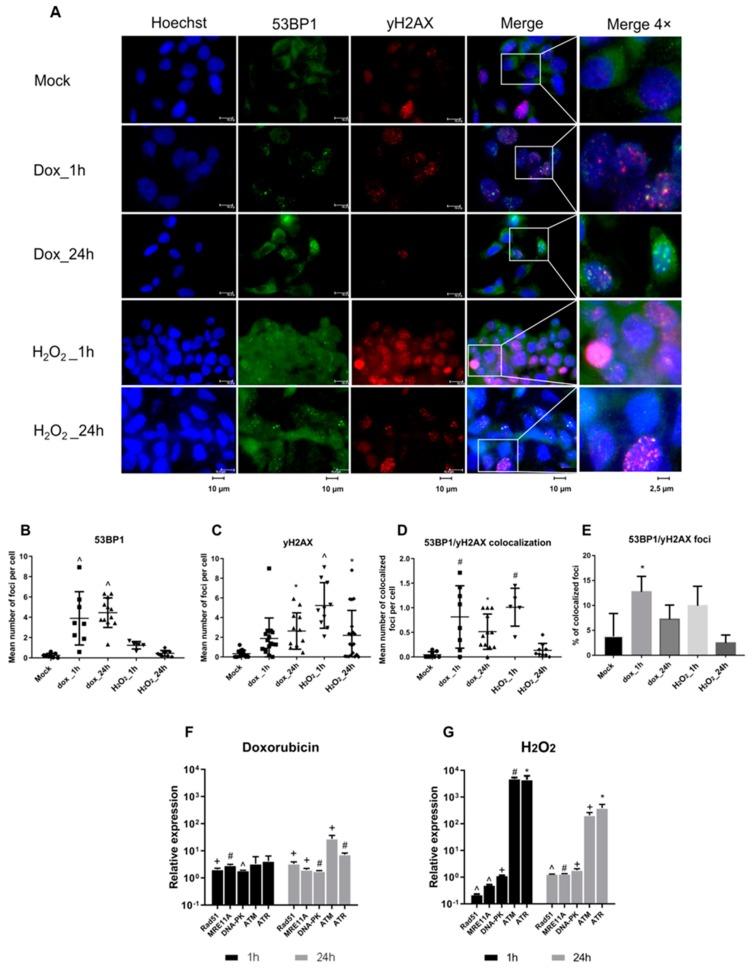
Study of DDR in response to doxorubicin and H_2_O_2_ treatment in HBV replication model. (**A**) Detection by immunofluorescence of the formation of 53BP1 (green) and yH2AX (red) foci 1 h and 24 h after treatment with doxorubicin (dox) or H_2_O_2_. Cell nuclei were counterstained with Hoechst33342 dye (blue). Quantitative analysis of (**B**) 53BP1 and (**C**) yH2AX foci, (**D**) the mean number of all co-localized 53BP1/yH2AX foci per cell, and (**E**) percentage of co-localized 53BP1/yH2AX foci calculated as the ratio of co-localized foci to the total number of both types of foci. Expression of DDR factors 1 h and 24 h post (**F**) doxorubicin and (**G**) H_2_O_2_ treatments. Asterisks indicate statistically significant differences in means compared to control. * *p* < 0.05, ^+^ *p* < 0.01, ^#^ *p* < 0.001, ^^^ *p* < 0.0001.

Doxorubicin and H_2_O_2_ induce DNA damage and DDR leads to the formation of yH2AX and 53BP1 foci [24]. We followed DDR signaling in the HepG2-1.1merHBV cell line treated with doxorubicin and H_2_O_2_ by registering the formation of yH2AX and 53BP1 using immunocytochemistry (ICC). For this, cells were treated with doxorubicin and H_2_O_2_ for 1 h and analyzed by ICC immediately or 24 h post treatment. Treating cells with doxorubicin and H_2_O_2_ inflicted severe damage of cellular DNA as indicated by generation of multiple yH2AX and 53BP1 foci (Figure 2A–C). Doxorubicin induced formation of multiple yH2AX and 53BP1 foci already 1 h pt; the mean number of both yH2AX and 53BP1 foci increased 24 h pt (Figure 2B). The yH2AX and 53BP1 foci were co-localized (Figure 2D,E). The latter is an accepted marker of the induction of double-stranded DNA breaks (DSBs) corroborating earlier findings [24]. In contrast to doxorubicin, H_2_O_2_ induced the formation of multiple, distinct yH2AX, but few 53BP1 foci (Figure 2A–C) which were not co-localized (Figure 2D,E). These data indicated that H_2_O_2_ mostly caused single-stranded DNA breaks (SSBs) (Figure 2D,E). Removing H_2_O_2_ resulted in the disappearance of a significant portion of both yH2AX and 53BP1 foci already 24 h pt indicating DNA repair. 

Formation of yH2AX and 53BP1 foci occurs shortly after DNA damage and is associated with the activity of PIKKs, namely ATM, ATR, and DNA-PKcs [16]. Using semi-quantitative PCR, we monitored the transcription of ATM, ATR, and DNA-PKcs together with RAD51 (a DDR factor) [36] and a factor involved in recognizing DNA damage MRE11A [37]. We found that treating cells with doxorubicin and H_2_O_2_ resulted in the significant upregulation of the levels of ATM and ATR mRNA, while transcription of other DDR factors remained basically unaltered. Doxorubicin and H_2_O_2_ induced over 10-fold upregulation of transcription of ATM and ATR, in case of H_2_O_2_ partially decreasing the day after the treatment (Figure 2F,G). 

Collectively, these results demonstrated that DNA damage caused by doxorubicin and H_2_O_2_ (doxorubicin inducing DSBs and H_2_O_2_, SSBs) leads to the upregulation of HBV transcription through a mechanism associated with the upregulation of ATM and ATR transcription. 

### 3.2. The Role of ATM and ATR in HBV Replication

**Figure 3:**
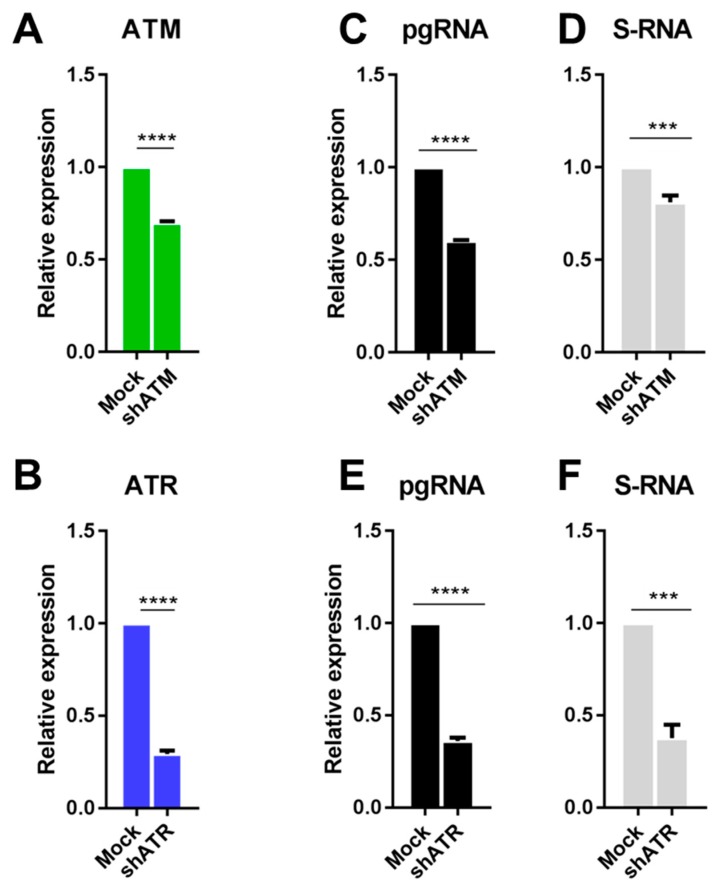
Downregulation of ATM and ATR diminishes HBV transcription. Downregulation of target gene transcription in HEK-293T cells transfected with shRNAs targeting (**A**) ATM and (**B**) ATR. Effects of downregulation of ATM (**A**) and ATR (**B**) on the levels of HBV (**C**,**E**) and S-RNA (**D**,**F**). The results were reproduced in at least 3 independent experiments. Asterisks indicate statistically significant differences in means compared to the control. *** *p* < 0.001, **** *p* < 0.0001.

To determine the role of individual DDR factors in HBV replication, we silenced the expression of ATM and ATR with shRNA (see experimental scheme in Figure S2). Initially, shRNAs were tested in HEK-293T cells transiently co-transfected with shRNA and an HBV-expressing plasmid. HEK-293T cells express SV40 large T antigen (SV40LT) which may mask DDR [21], still, they present a useful test system for primary assessment of the effects of shRNAs on HBV replication. ShRNA against ATM (shATM) downregulated the expression levels of ATM by 20% (Figure 3A), whereas shRNA against ATR (shATR) completely abolished ATR transcription (Figure 3B). Interestingly, partial knockdown of ATM transcription reduced HBV pgRNA by over 40% (Figure 3C) and S-RNA by 10% (Figure 3D), while abolishment of transcription of ATR reduced it by ~50–60% (Figure 3E,F). 

**Figure 4:**
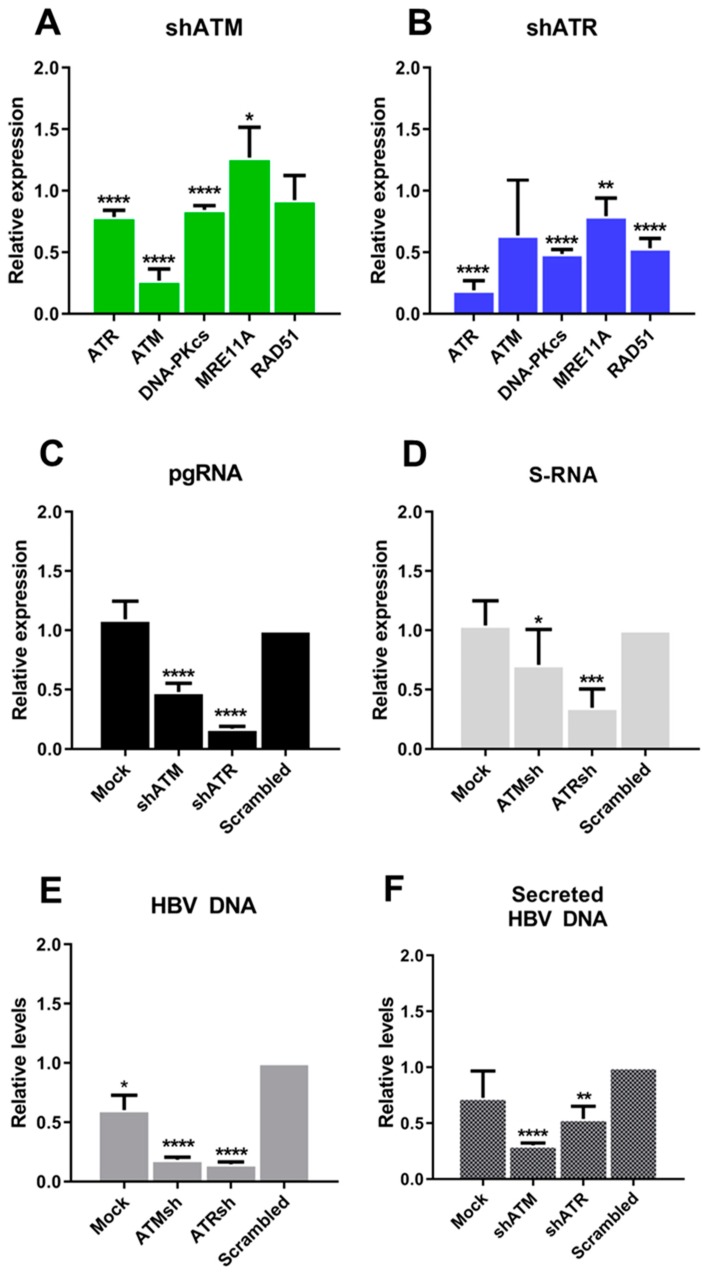
Suppression of HBV replication by ATM and ATR knockdown. Alterations in the transcription of DDR factors in HepG2-1.1merHBV cells transfected with (**A**) shRNA against ATM and (**B**) shRNA against ATR. Knocking down ATM (shATM) and ATR (shATR) reduces (**C**) HBV pgRNA, (**D**) HBV S-RNA, (**E**) intracellular HBV DNA levels and (**F**) secreted HBV DNA levels. The results were reproduced in at least 3 independent experiments. Asterisks indicate statistically significant differences in means compared to control. * *p* < 0.05, ** *p* < 0.01, *** *p* < 0.001, **** *p* < 0.0001.

Next, we analyzed the effects of ATM and ATR knockdowns in the HepG2-1.1merHBV cell line. In this cell line, the transfection of shATM or shATR reduced target gene transcription by ~80% (Figure 4A,B). Knocking down ATM also slightly decreased the levels of transcription of ATR and MRE11A (Figure 4A), whereas knocking down ATR significantly decreased the expression of ATM, DNA-PKcs, and RAD51 mRNAs (Figure 4B; compared to scrambled control). Both shATM and shATR reduced the levels of HBV pgRNA (Figure 4C), HBV S-RNA (Figure 4D), intracellular HBV DNA (Figure 4E) and secreted HBV DNA (Figure 4D). ShATR also reduced the intracellular levels of HBV DNA, secreted HBV DNA and pgRNA by 50–85%. Similarly, shATM reduced intracellular and secreted HBV DNA levels by ~80% and pgRNA levels by ~50%. Effects of shRNAs on S-RNA expression were less pronounced.

These data indicate that ATM and ATR play important roles in HBV replication in vitro. 

### 3.3. Transcriptional Activation of ATM and ATR Promotes HBV Replication

Since the elevation of ATM and ATR expression by DNA damage appears to be important for HBV replication, we aimed to find whether transcriptional activation of ATM and ATR is sufficient to promote HBV replication (see experimental scheme in Figure S3). 

**Figure 5:**
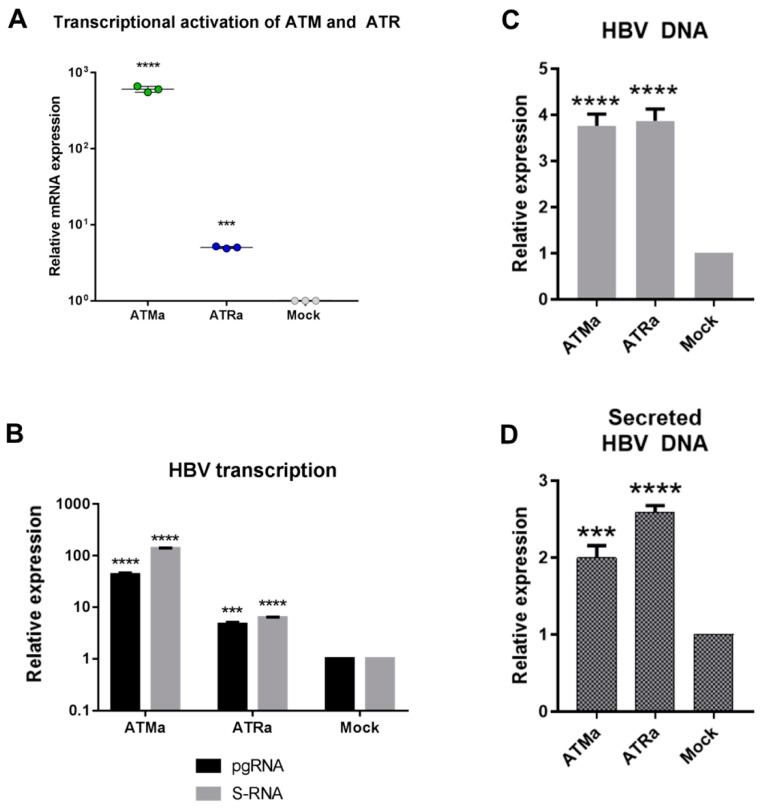
Increased ATM and ATR transcription stimulate HBV replication. (**A**) mRNA levels of ATM or ATR upon transcriptional activation of ATM (ATMa) or ATR (ATRa), respectively. ATM and ATR mRNA levels were normalized to a mock-treated control. Effects of ATMa and ATRa on (**B**) HBV transcription, and the levels of (**C**) intracellular and (**D**) secreted HBV DNA. The results were reproduced in at least 3 independent experiments. Asterisks indicate statistically significant differences in means compared to the control. *** *p* < 0.001, **** *p* < 0.0001.

In a dCas9-p300 variant of CRISPRa technology, a catalytically inactive Cas9 (dead Cas9; dCas9) linked to a strong activator of transcription p300 (catalytic subunit of p300 acetyltransferase) adds marks of active chromatin to the regulatory regions of genes and turns on gene transcription [29]. We designed sgRNAs targeting dCas9-p300 protein regulatory elements of ATM and ATR genes. Transfection of CRISPRa into HepG2 cells enhanced ATM levels >600-fold and ATR levels >4-fold compared to a mock-treated control (Figure 5A). In HepG2 cells co-transfected with CRISPRa and HBV recombinant cccDNA, CRISPRa-mediated activation of ATM or ATR transcription greatly induced HBV replication. Overexpression of ATM resulted in a 40–140-fold increase in HBV transcription (Figure 5B), ~4-fold increase in intracellular HBV DNA levels (Figure 5C) and ~2-fold increase in the levels of secreted HBV DNA (Figure 5D). Overexpression of ATR was also associated with increased HBV replication, although to a lesser extent than the overexpression of ATM. It led to a ~6-fold increase in HBV transcription (Figure 5B), ~4-fold increase in intracellular HBV DNA (Figure 5C) and secreted HBV DNA levels (Figure 5D). Experiments with shRNAs demonstrated that ATR affects HBV replication more potently than ATM. Here, a smaller increase of HBV replication due to transcriptional activation of ATR compared to ATM could have been due to the lower efficacy of the former. 

## 4. Discussion

Earlier studies demonstrated that replication of HBV in human cells results in DNA damage inflicted by several independent but overlapping mechanisms [38,39,40]. Thirty years ago Capovilla A et al., demonstrated that HBV X-protein binds to damaged DNA and sensitizes liver cells to ultraviolet irradiation [41]. Further studies revealed that it interferes with the repair of cellular DNA by inhibiting the repair of nucleotide excisions [42,43]. DDR pathways were apparently exploited by HBV for replication and reactivation [14,20,44]. The key kinases activated in response to DNA damage are ATM and ATR [16]. Zhao F et al. had shown that HBV infection triggers DDR ATR signaling, while the downregulation of ATR expression reduces HBV replication [25]. In spite of this finding, the actual roles of ATM, ATR, and other DDR factors in HBV replication and reactivation remain largely unknown.

Doxorubicin and H_2_O_2_ induce DNA damage. The severity of the overall DNA damage they induce is similar, as reflected in our study by the close total number of yH2AX and 53BP1 foci generated in cells after each of the treatments (Figure 2A–E). Here, we have shown that both treatments strongly upregulate the transcription of ATM and ATR, while relative transcription levels of other DDR factors, such as DNA-PKcs, MRE11A, or RAD51, remain basically unaffected. Doxorubicin and H_2_O_2_ cause DNA damage of different types. Doxorubicin induces DNA DSBs characterized by co-localized yH2AX and 53BP1 foci which trigger ATM signaling [45]. H_2_O_2_ rarely induces DSBs, but generates numerous DNA SSBs characterized by multiple yH2AX and rare 53BP1 foci, which trigger ATR signaling [45] (Figure 2A–E). Both types of DNA damage, by doxorubicin and H_2_O_2_, significantly elevated HBV replication. However, SSBs caused by H_2_O_2_ activated ATM and ATR transcription more efficiently and resulted in higher levels of HBV intermediates than DSBs induced by doxorubicin. These results strengthen the role of DNA damage response and ATM and ATR signaling in HBV reactivation.

Kim et al. had shown that HBx of HBV induces yH2AX formation and activates ATM signaling and associated these findings with HBV pathogenesis [26]; the actual effects of ATM on HBV replication were not evaluated. Here, in in vitro systems (HepG2, HEK-293T, HepG2-1.1merHBV, HepG2-1.5merHBV) we have shown that the downregulation of transcription of ATR and ATM by shRNA reduced HBV transcription, and intracellular and secreted HBV DNA levels by 50–80%, confirming the role of ATM and ATR signaling in HBV replication. The effects on HBV replication of ATR knockdown were more pronounced than of ATM, which corroborated the findings of Zhao et al. [25]. To dissect the actual role of ATM and ATR in the HBV life cycle, we activated the transcription of both kinases by CRISPRa technique. Transcriptional activation of ATM or ATR markedly increased the levels of HBV pgRNA, HBV S-RNA, intracellular and secreted HBV DNA, confirming their role in HBV replication/reactivation, namely in enhancing HBV transcription and replication upon DNA damage. Unfortunately, we could not compare the potency of HBV reactivation by ATM and ATR due to the difference in the efficacy of ATM and ATR transcriptional activation by CRISPRa. 

DDR is a specific and hierarchical network that includes cell cycle checkpoints, DNA repair, DNA-damage tolerance and alterations in cell metabolism [46]. Here, we modelled HBV replication in the transformed cells (HEK293T, HepG2, HepG2-1.1merHBV, HepG2-1.5merHBV), in which these mechanisms could malfunction [47]. In this respect, they may not adequately represent the primary hepatocytes. However, the triggering of HBV replication in such cells by DNA damage and DDR signaling indicates that in the liver such cells can serve as a depo for active viral production. The actuality of the DNA damage mechanism of HBV (re)activation for the cells with the intact DDR would constitute the subject of a separate study, which would require a rigorous analysis of the interactions between DNA-damage, ATM/ATR, and HBV in physiologically relevant conditions.

Previous large-scale studies demonstrated that ATM and ATR kinases phosphorylate hundreds of substrates and initiate widespread cell responses [18]. Following DNA damage, ATM and ATR rapidly reprogram transcriptional and post-transcriptional profiles of the affected cells [48,49]. There is evidence that ATM/ATR may be involved in epigenome remodeling (HDAC1/4, EZH2), cell cycle checkpoint signaling, regulating gene transcription, controlling RNA stability, and other processes [16,50,51,52,53]. These events underlie the role of ATM and ATR signaling in viral replication demonstrated for herpesviruses [54,55], papillomaviruses [56,57], and polyomaviruses [58,59]. Here, our data indicate that ATM and ATR signaling is an important host factor involved in HBV replication, specifically in triggering HBV reactivation. The molecular mechanism(s) of the pro-viral activity of ATM and ATR signaling for HBV as well as for the other viruses have yet to be elucidated. 

In the era of direct-acting antivirals, HBV reactivation can be successfully managed in clinical practice [60,61]. Still, it can be a severe and often fatal complication in patients with an overt or occult HBV infection or HBV-associated cancer. HBV reactivation depends on many factors, such as the immune status of patients [5], HBV mutations [62], co-infection with other viruses [63], and/or additional therapies as the ones administered to patients with HBV-associated cancer [5,6,64]. Our finding that HBV replication and HBV reactivation may be induced by transcriptional activation of ATM and ATR (ATM-ATR signaling) in response to DNA damage, points to the necessity of carefully choosing drugs administered to HBV-infected patients to avoid ones with DNA-damaging activity.

To conclude, HBV replication is tightly connected to DDR, namely ATM and ATR signaling. Activation of the latter by DNA-damaging agents may drive HBV reactivation. Further analysis of DDR signaling at the protein level and testing of known drugs and novel compounds for the capacity to suppress ATM/ATR signaling and concomitant HBV replication are urgently needed. 

## Figures and Tables

**Table 1:** Concentrations of doxorubicin and H_2_O_2_ used in the study.

**Agent**	**Number**	**Concentration (mM)**
H_2_O_2_	1	0.01
	2	0.4
	3	2
	4	3
	5	4
	6	7
	7	8
	8	9
**Agent**	**Number**	**Concentration (μM)**
Doxorubicin	1	0.1
	2	0.2
	3	1.5

**Table 2:** Primers and probes used in the study.

Target	Sequence	
GAPDH mRNA	fw	5′-CCAGGTGGTCTCCTCTGACTT-3′
	rev	5′-GTTGCTGTAGCCAAATTCGTTGT-3′
	probe	FAM-AACAGCGACACCCACTCCTCCACC-BHQ1
cссDNA	fw	5′-CCGTGTGCACTTCGCTTCA-3′
	rev	5′-GCACAGCTTGGAGGCTTGA-3′
	probe	FAM-CATGGAGACCACCGTGAACGCCC-BHQ1
pgRNA	fw	5′-GGTCCCCTAGAAGAAGAACTCCCT-3′ [35]
	rev	5′-CATTGAGATTCCCGAGATTGAGAT-3′ [35]
	probe	FAM-TCTCAATCGCCGCGTCGCAGA-BHQ1 [35]
S-RNA	fw	5′-TCCTCCAAСTTGTCCTGGTTATC-3′ [35]
	rev	5′-AGATGAGGCATAGCAGCAGGAT-3′ [35]
	probe	FAM-ATGATAAAACGCCGCAGACACATCCAGC-BHQ1 [35]
HBV DNA and β-globin		АmpliSens^®^ HBV monitor (CRIE)
ATM mRNA	fw	5′-AGTTTCATCTTCCGGCCTCT-3′
	rev	5′-GCTGTGAGAAAACCATGGAAG-3′
ATR mRNA	fw	5′-AACATTCGTGGCATTGACTG-3′
	rev	5′-AAGCAAGGTGATCTCATCCG-3′
PRKDC mRNA	fw	5′-CTTACATGCTAATGTATAAGGGCG-3′
	rev	5′-CAGCAGGCACTTTACTTTCTC-3′
MRE11A mRNA	fw	5′-TCAGTTAGGTGGGTCTGGGT-3′
	rev	5′-AGCGGTGAACTGAATCGCAT-3′
RAD51 mRNA	fw	5′-TCACGGTTAGAGCAGTGTG-3′
	rev	5′-AACAGCCTCCACAGTATGG-3′

## Supplementary Materials

The following are available online at https://www.mdpi.com/1999-4915/11/11/997/s1, Figure S1: Experimental schematics; Figure S2: Experimental schematics for shRNAs transfections; Figure S3: Experimental schematics for transcriptional activation of ATM and ATR.
