# Supplementary material for: ATM and ATR Expression Potentiates HBV Replication and Contributes to Reactivation of HBV Infection upon DNA Damage
Source: Viruses. 2019 Oct 31;11(11):997. doi: 10.3390/v11110997 (PMC6893526; doi:10.3390/v11110997)
Supplement: Supplementary file 1 [file viruses-11-00997-s001.pdf]

ATM and ATR Expression Potentiates HBV Replication and Contributes to Reactivation of HBV Infection upon DNA Damage

Kostyusheva A.[1], Brezgin S.[1,2], Bayurova E.[3,4], Gordeychuk I.[3,4,5], Isaguliants M.[3,4,6,7], Goptar I.[8], Urusov F.[8], Nikiforova A.[8], Volchkova E.[5], Kostyushev D.[1] and Chulanov V.[1,5,9]

Supplementary materials

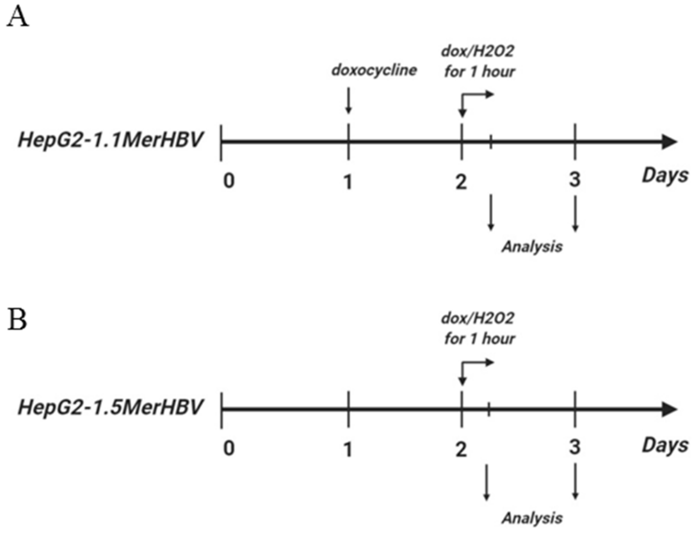

Figure S1. Experimental schematics. (A) HepG2-1.1merHBV were first treated with doxycycline for 24 h to induce HBV expression then incubated with doxorubicin or H2O2 solutions for 1 h. Treated and untreated cells were harvested immediately or 24 h pt. (B) HepG2-1.5merHBV cells were treated by doxorubicin or H2O2 solutions for 1 h and harvested immediately or 24 h pt.

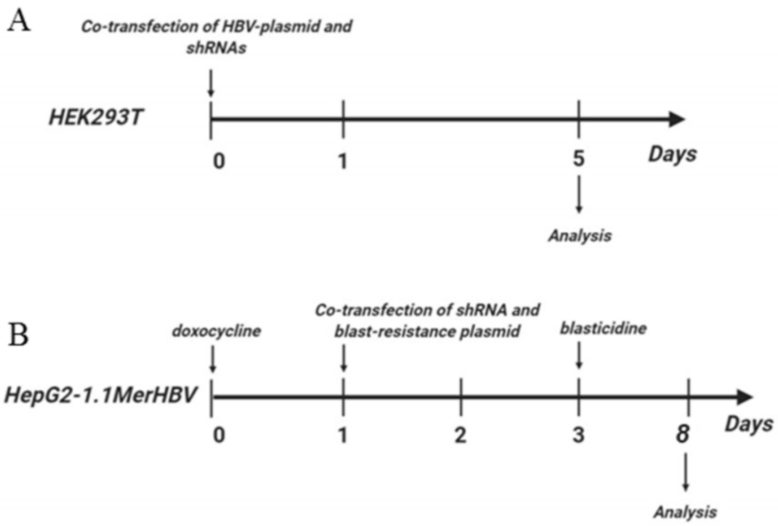

Figure S2. Experimental schematic for shRNAs transfections. (A) HEK293T cells were co-transfected with HBV-encoding plasmid and an shRNA-encoding plasmid using polyethylenimine; cells were harvested for analysis 5 days pt. (B) HepG2-1.1merHBV cells were treated by doxycycline for 24 h to induce HBV expression, co-transfected by shRNA-encoding plasmid and blasticidine-resistance plasmid the following day using Lipofectamine 3000. Transfected cells were selected with blasticidine for 5 days and harvested for analysis.

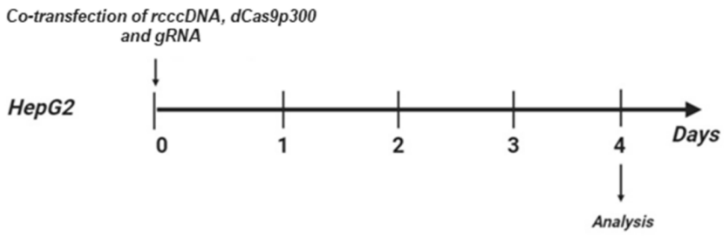

Figure S3. Experimental schematic for transcriptional activation of ATM and ATR. HepG2 cells were co-transfected by a recombinant cccDNA, dCas9p300 and a gRNA targeting either ATM or ATR promoter using Lipofectamine 3000 and harvested at day 4 pt.
